# Development and internal validation of a Nomogram for preoperative prediction of surgical treatment effect on cesarean section diverticulum

**DOI:** 10.1186/s12905-019-0817-z

**Published:** 2019-11-11

**Authors:** Yizhi Wang, Qinyi Zhu, Feikai Lin, Li Xie, Jiarui Li, Xipeng Wang

**Affiliations:** 1 Department of Gynecology & Obstetrics, Xinhua Hospital affiliated to Shanghai Jiao Tong University School of Medicine, 1665 Kong Jiang Road, Shanghai, 200092 China; 2 Shanghai first maternity and infant health institute, Shanghai, China; 3 Department of Gynecology & Obstetrics, Xinhua Hospital affiliated to Shanghai Jiao Tong University School of Medicine, Shanghai, China; 4 Clinical Statistics Center, Fudan University Shanghai Cancer Center, Shanghai, China

**Keywords:** Cesarean section diverticula, Cesarean scar defect, Thickness of remaining muscular layer, Nomogram, Vaginal repair

## Abstract

**Background:**

The aim of this study was to develop and validate an individualized score based on preoperative parameters to predict patient outcomes after vaginal repair of cesarean section diverticulum.

**Methods:**

This is a retrospective cohort study (Canadian Task Force classification II-2). Patients were enrolled between Jun 11, 2012, to May 27, 2016. Multivariable logistic regression analyses were used to construct the predictive model. Then, we generated a nomogram to assess the individualized risk of poor prognosis after operation. This prediction model included information from 167 eligible patients diagnosed with cesarean section diverticulum who underwent vaginal repair. Class-A healing group was defined as CSD patients who had menstruation duration of no more than 7 days and a thickness of the remaining muscular layer of no less than 5.8 mm after vaginal repair according to conferences. Others were included in the non-class-A healing group. A final nomogram was computed using a multivariable logistic regression model.

**Results:**

The factors contained in the individualized prediction nomogram included the depth/ the thickness of the remaining muscular layer ratio, number of menstruation days before surgery, White blood cell and fibrinogen. This model demonstrated adequate discrimination and calibration (C-index = 0.718). There was a significant difference in the number of postmenstrual spotting days (12.98 ± 3.86 VS 14.46 ± 2.86, *P* = 0.022) and depth/ the thickness of the remaining muscular layer ratio (2.81 ± 1.54 VS 4.00 ± 3.09, *P* = 0.001) between two groups. Decision curve analysis showed that this nomogram was clinically useful.

**Conclusions:**

This cesarean section diverticulum score can predict the outcomes of cesarean section diverticulum and can be useful for counseling patients who are making treatment decisions.

## Text

### Background

Over the last several decades, the incidence of delivery via cesarean section (C-section) has increased around the world [1, 2]. Approximately two-thirds of women in Chinese cities selected cesarean section delivery between 1990 and 2002 [3]. An investigation of 39 hospitals in China indicated that the incidence of C-Section without indication was 24.553% [4]. It has been reported that many patients who underwent C-section developed C-section scar diverticulum (CSD) after surgery [5]. The prevalence of a niche ranged from 24 to 70% when assessed by transvaginal sonography [6] and 19.4 to 88% according to symptom [7]. The correlation between number of C-section and increased risk of CSD hasn’t been figured out yet though, only few references considered multiple CSs as probable risk factors [6]. CSD can result in long-term complications, such as prolonged menstrual bleeding (the menstrual cycle is more than seven days), C-section scar ectopic pregnancy, dyspareunia, dysmenorrhea and chronic pelvic pain [8, 9]. Accumulation of blood in the cesarean scar defect can cause inflammation, influence the mucus quality and make an adverse environment for embryo implantation. As a result, patients suffered the pain of secondary infertility [10–12]. Moreover, C-section scar ectopic pregnancy can increase the incidence of uterine scar rupture, which threatens both the life of the neonate and mother [13]. Furthermore, many reports have demonstrated that postmenstrual bleeding caused by CSD is the most typical manifestation, which severely affects the quality of life of patients [14, 15].

No clinical guidelines have been issued for the treatment of CSD based on the thickness of the remaining muscular layer (TRM) or/and prolonged menstrual bleeding. Surgical treatment is a reasonable management approach for CSD since medical therapy is not consistently effective. Many surgical treatments have been reported [16, 17], such as endometrial ablation [18]. hysteroscopic surgery [19], vaginal surgery [1], and laparoscopic surgery [13, 20]. In previous study, we reported that vaginal repair of CSD is a very effective surgery for repairing anatomical defects and reducing the number of menstruation days [21]. Though Tulandi’s meta-analysis quotes menstruation days improvement in 89 to 93.5% of patients with CSD after vaginal repair surgery [22], however, only 28.2% of cases experienced a reduction in the number of menstruation days to less than 7 according the previous study [21]. The results confirmed that many CSD patients are difficult to cure, which severely affects the quality of life of women with CSD. The thickness of the remaining muscular layer (TRM) of CSD patients is considered to be the most important factor for determining subsequent pregnancy safety related to C-section scar ectopic pregnancy, uterine scar rupture and other complications [23, 24].

The surgical curative effects of CSD are difficult to evaluate because there are many potential risk factors for CSD. These risk factors include the multiple cesarean sections, retroflexed uteri [25], technique for repairing the uterine incision during cesarean section [26] and other factors [7]. Our study was the first to report the use of uterine contrast-enhanced Magnetic Resonance Imaging (MRI) for CSD evaluation [27]. MRI is usually used for a preoperative work-up, and uterine contrast-enhanced MRI is a much better imaging method to measure the TRM, length, width and depth of the CSD than a general MRI scan. Although MRI assessment has been shown to be useful for patients with CSD, an optimal approach that combines multiple biomarkers as predictive factors has not been found yet.

Therefore, the aim of this study was to develop and validate an individualized score for preoperative prediction of outcomes in patients with CSD.

## Methods

### Patients

Between Jun 11, 2012, and May 272,016, 228 Chinese women underwent vaginal repair for CSD at Shanghai First Maternity and Infant Hospital, Tongji University School of Medicine. These women all had prolonged postmenstrual spotting and underwent treatment in our hospital. The research protocol was approved by the relevant Institutional Review Board before the study began. This study was approved by the Ethics Committee of Shanghai First Maternity and Infant Hospital, affiliated with Tongji University (KS1512), and was conducted in accordance with the Declaration of Helsinki.

We reviewed and collected the patients’ medical records and follow-up data after they provided informed consent. All participants gave written informed consent before the study began. The author(s) agreed to provide copies of the appropriate documentation if requested. Baseline clinicopathologic data, including delivery times, menstrual cycle, age, gravidity, parity, age at first C-section, number of C-sections, hemoglobin (Hb) and data from MRI imaging, were also recorded before surgery. Laboratory analysis of Hb was conducted via a regular blood test within 3 days of surgery.

Patients treated by vaginal surgery were included in the study with the following criteria: 1) clinical features, such as longer menstruation after C-section and no significant change in the menstrual cycle; 2) history of C-section; and 3) CSD detected by MRI. Exclusion criteria included uterine pathologies, such as adenomyosis, leiomyoma and other conditions [21].

### Procedure for vaginal repair of CSD

Each patient received continuous epidural anesthesia while in the lithotomy position. At a distance of 0.5 cm below the site of the reflexed vesicocervical area,an anterior incision was made from the 3 o’clock position to the 9 o’clock position using an electric knife. The bladder was carefully dissected away from the uterus with sharp dissection scissors toward the abdominal cavity until the peritoneum was reached. Once the abdominal cavity was entered and the cervical and lower uterine segments were exposed. The CSD tissue was cut to the normal healthy muscle. The incision was closed with a double layer of 1–0 absorbable interrupted sutures. After adequate hemostasis, the peritoneum and bilateral bladder column were sutured, followed by the incision in the cervical vaginal area.

### Follow-up

**Fig. 1:**
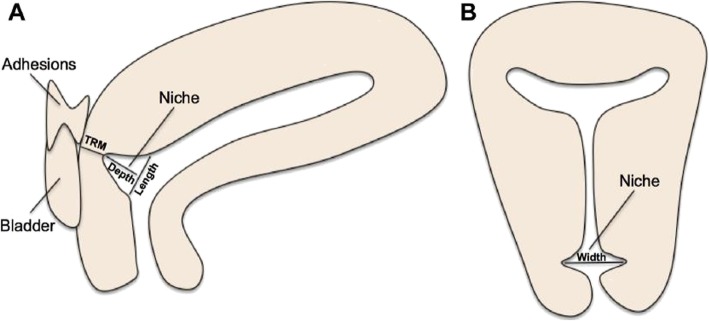
MRI measurements [21]

Patients included in the study had follow-up clinic visits to record their menstruation at 1, 3 and more than 6 months after the procedure and measure the CSD scar site by MRI at more than 6 months after the procedure. According to the previous study, patients’ menstruation would likely plateau at follow-up visits more than 3 months after surgery [21]. The data from MRI were evaluated at the same center by an experienced radiologist. The data after surgery mainly included the number of menstruation days and the depth, length, width, and thickness of the remaining muscular layer (TRM) as well as the depth/ TRM ratio based on contrast-enhanced MRI [21](Fig. 1). Primary outcomes were the number of postmenstrual spotting days and depth/ TRM ratio. All events and any modifications that occurred during follow-up were recorded.

We defined the Class-A healing group as CSD patients who had menstruation duration of no more than 7 days and a thickness of the remaining muscular layer of no less than 5.8 mm after vaginal repair, and all other patients were included in the non-class-A healing group [28].

### Statistical analysis

Patient characteristics and preoperative factors were analyzed using student’s t test and chi-square tests. Ages are given as the medians with ranges, others variables are expressed as mean ± SD. Multivariate logistic regression models were used to assess risk factors associated with non-class-A healing of CSD. Regression coefficients were used to generate prognostic nomograms. Model discrimination was measured quantitatively with the concordance index. Internal validation was performed using 1000 bootstrap resampling to quantify the overfitting of our modeling strategy and predict future performance of the model.

We incorporated both the depth/TRM ratio measured by MRI and clinical factors into a personalized nomogram for facilitating preoperative prediction of non-class-A healing in CSD patients. Multimarker analyses have been used in recent years for incorporating individual factors into marker panels [29].

All statistical analyses were performed by R software (version 3.3.2). The statistical significance levels were two-sided, with a *P* value of .05 or less.

### Developing a prediction model

Multivariable logistic regression analysis was used to assess the individualized prediction model with the following clinical candidate factors taken before surgery: the depth/ TRM ratio via MRI, number of menstruation days after C-section, WBC and fibrinogen. We built the final nomogram based on logistic regression analysis in the training cohort.

### Performance of the Nomogram in the training cohort

A calibration curve was plotted to evaluate the calibration of the nomogram using the Hosmer-Lemeshow test. A significant test statistic indicated that the prediction model did not calibrate perfectly [30]. Harrell’s C-index was computed to quantify the performance of the nomogram.

### Internal validation of the Nomogram

Internal validation was carried out using data from 167 patients.

### Clinical use

Decision curve analysis was performed to determine the clinical usefulness of the nomogram by quantifying the net benefits at different threshold probabilities.

## Results

### Patient characteristics

**Fig. 2:**
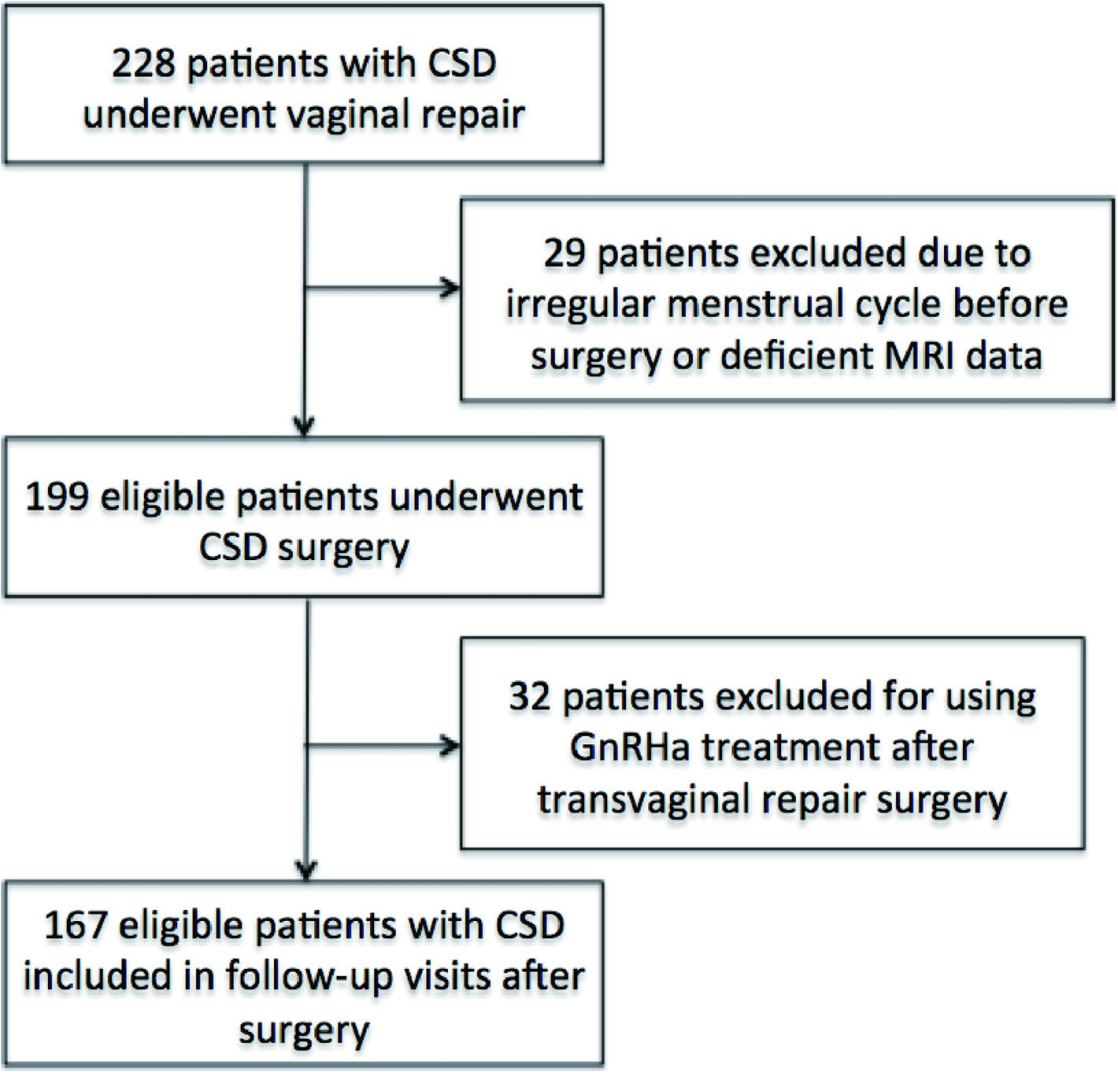
Flow chart of the study population. 167 patients were finally included in the cohort

Overall, 228 patients who presented with prolonged menstrual bleeding or very thin TRM due to CSD underwent vaginal repair between Jun 11, 2012, and May 27, 2016. Sixty-one of the patients were excluded from the analysis because of an irregular menstrual cycle prior to C-section, deficient MRI data or GnRHa treatment after the transvaginal repair surgery. Finally, 167 patients were included in the training cohort and assigned to record their menstruation and have their CSD scar site measured by MRI (Fig. 2).

**Fig. 3:**
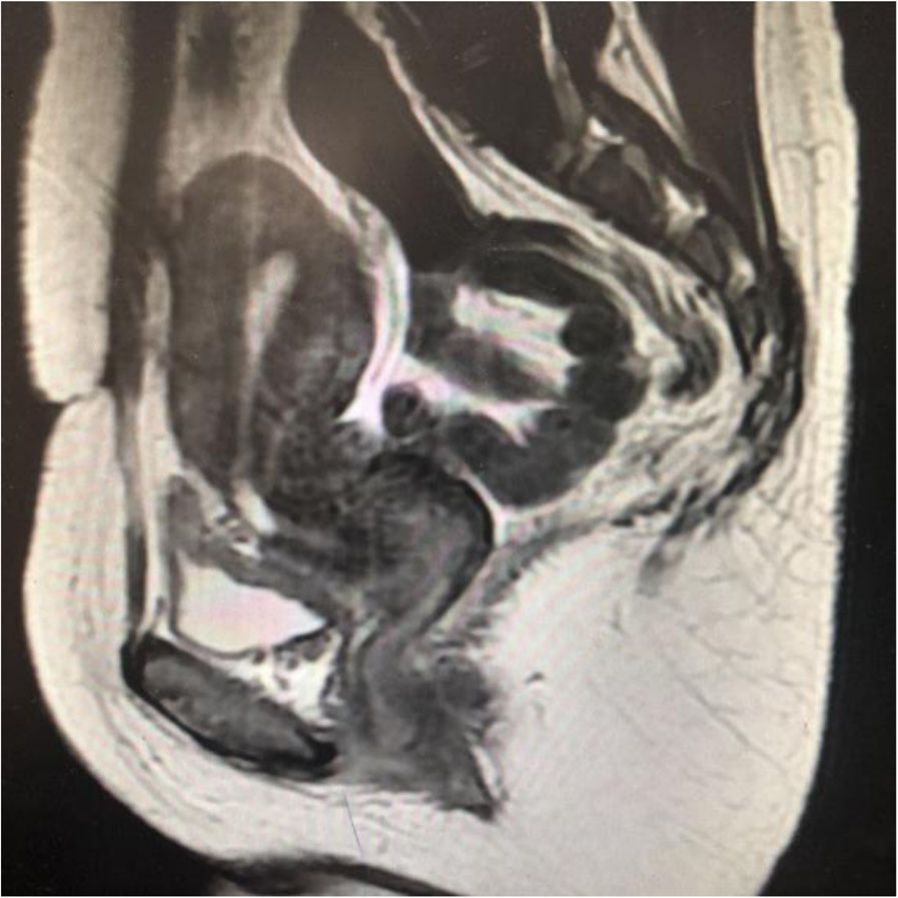
Preoperative picture via MRI

**Fig. 4:**
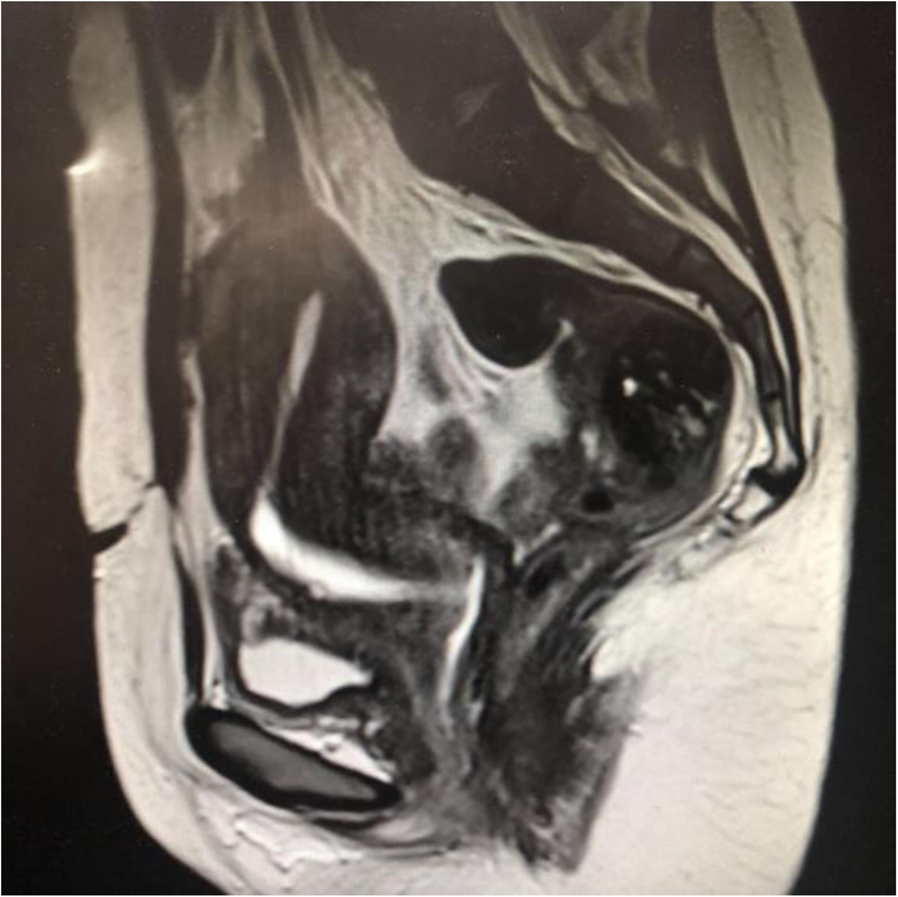
Postoperative picture via MRI

The patient characteristics of the training cohort are summarized in Table 1. The median age of the study patients was 32(range: 23 to 41 years). All of these women received one or two C-sections prior to the procedure. In the whole cohort, all of the patients presented with median postmenstrual spotting of 14.06 ± 3.21 days before vaginal repair surgery. The median thickness of the remaining muscular layer measured via MRI before vaginal repair surgery was 2.51 ± 1.12 mm. The median depth via MRI was 7.32 ± 2.95 mm (Table 1). After surgery, postmenstrual spotting days shortend significantly (8.48 ± 2.35 VS 14.06 ± 3.218, *P* <  0.001) (Table 2) (Figs. 3 and 4).

**Table 1:** Characteristics of Patients in the Training Cohorts

Characteristic	Class-A Healing	Non-Class-A Healing	*P* value
All CSD patients (*n* = 167)	*n* = 45	*n* = 122	
Age, years	33(26–41)	32(23–41)	0.211
Age at first C-section, years	28(19–34)	27(21–36)	0.372
Numbers of C-section			0.930
*n* = 1	32	85	
*n* ≥ 2	13	37	
Postmenstrual spotting before sugery, days	14.06 ± 3.21	8.48 ± 2.35	< 0.001
Postmenstrual spotting after sugery, days	6.36 ± 0.95	9.26 ± 2.22	< 0.001
MRI Length, mm	9.80 ± 3.40	10.20 ± 3.69	0.518
MRI Width, mm	18.50 ± 4.27	18.10 ± 4.82	0.622
MRI Depth, mm	6.23 ± 2.28	7.72 ± 3.07	0.003
MRI TRM, mm	2.54 ± 0.97	2.49 ± 1.18	0.816
The ratio Depth/TRM	2.81 ± 1.54	4.00 ± 3.09	0.001
Hb, g/L	122.16 ± 9.91	117.02 ± 21.17	0.035
WBCs, 10^9/L	5.83 ± 1.45	5.45 ± 1.50	0.146
Fibrinogen, g/L	2.12 ± 0.32	2.12 ± 0.48	0.930
Platelet, 10^9/L	233.17 ± 52.61	234.43 ± 58.03	0.899
Prothrombin time, PT	11.35 ± 1.66	11.74 ± 1.18	0.094

Age are given as the medians with ranges, others are given as mean ± SD

Blood test and MRI were taken before surgery in Table 1

**Table 2:** Comparison of CSD parameters and menstrual duration before and after vaginal repair in CSD patients

Characteristic	Before operation	After operation	*P* value
All CSD patients (*n* = 167)			
Days of postmenstrual spotting	14.06 ± 3.21	8.48 ± 2.35	< 0.001
MRI Length, mm	10.09 ± 3.61	5.12 ± 4.41	< 0.001
MRI Width, mm	18.21 ± 4.67	9.21 ± 6.16	< 0.001
MRI Depth, mm	7.32 ± 2.95	3.75 ± 2.46	< 0.001
MRI TRM, mm	2.51 ± 1.12	4.72 ± 2.17	0.003

Data are given as mean ± SD

There was a significant difference in the number of postmenstrual spotting days before vaginal repair surgery between class-A healing 12.98 ± 3.86 and non-class-A healing 14.46 ± 2.86 patients in the training cohort (*P* = 0.022). Moreover, the depth/ TRM ratio measured via contrast-enhanced MRI between class-A healing and non-class-A healing patients in the training cohort was also different: non-class-A healing patients generally had a higher ratio than class-A healing patients (4.00 ± 3.09 and 2.81 ± 1.54, respectively; *P* = 0.001) (Table 1). The depth/TRM ratio measured via MRI scanning combined multiple individual clinical factors showed adequate discrimination in the primary cohort (C-index, 0.718).

### Developing an individualized prediction model

**Fig. 5:**
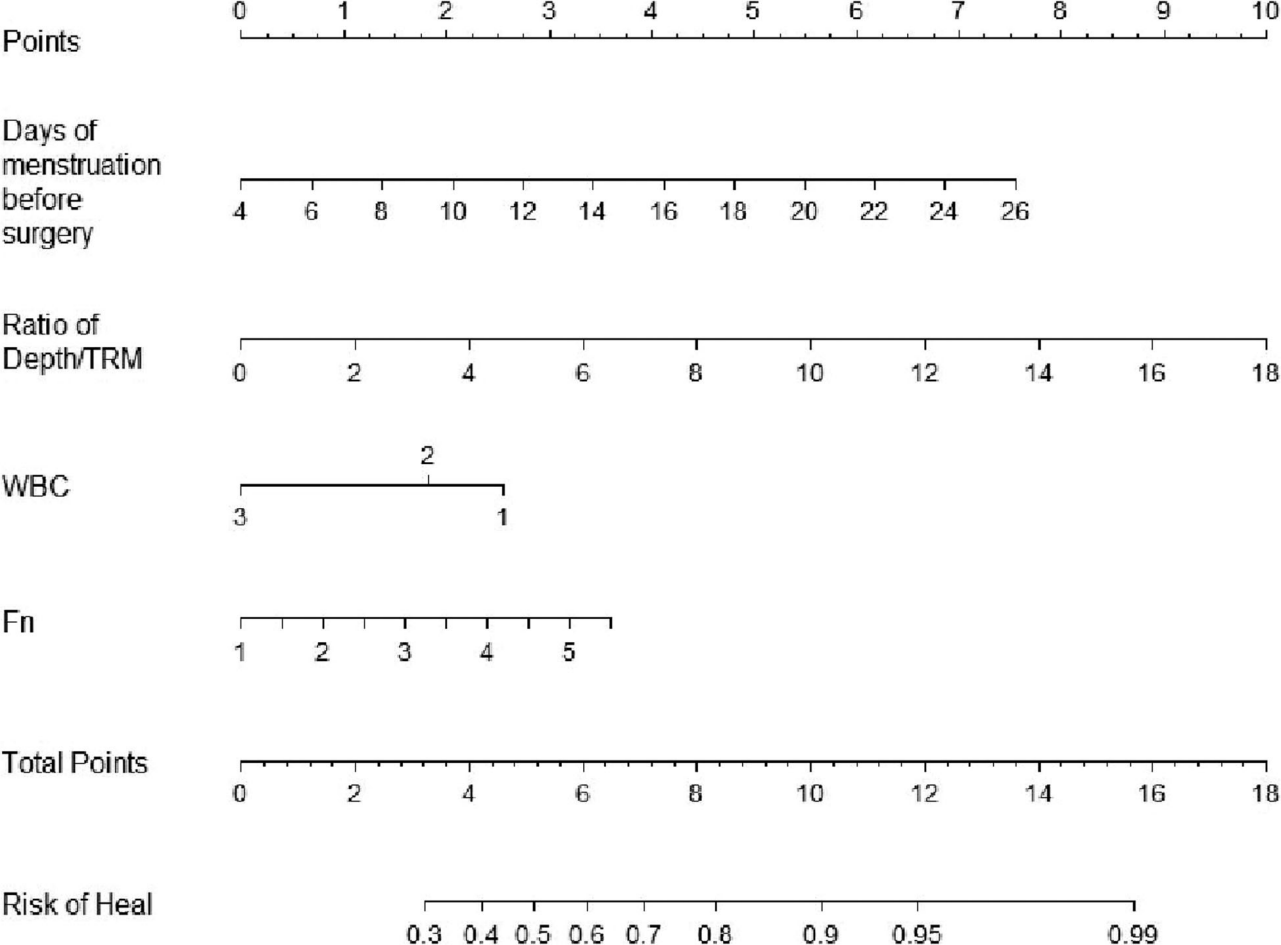
The developed nomogram. The nomogram was developed in the training cohort, with the days of menstruation before surgery, ratio of depth/TRM, WBC and fibrinogen. The model that was derived from β-regression coefficients of Logistic regression

Based on multivariate cox regression analysis, the depth/TRM ratio measured via MRI (OR, 1.275; 95% CI, 1.041 to 1.560; *P* = 0.019) and the number of menstruation days before surgery (OR, 1.162; 95% CI, 1.025 to 1.316; *P* = 0.090) of the 5 variables listed in Table 3 were associated with non-class-A healing in the training cohort. WBCs≤4.76 and 4.76 ≤ WBCs≤6.09 were also associated with an increased risk of non-class-A healing, and the odds ratios were 3.043 (95% CI, 1.195 to 7.750; *P* = 0.020) and 2.219 (95% CI, 0.930 to 5.295; *P* = 0.072), respectively. Additionally, lower level of fibrinogen was associated with an increased risk of non-class-A healing, with an OR of 1.419 (95% CI, 0.624 to 3.225; *P* = 0.404) (Table 3). The model that was derived from the estimated β-regression coefficients of these four variables was developed as a nomogram (Fig. 5).

**Table 3:** Risk Factors for Non-class-A Healing in Patients with CSD

Variables	β coefficient	S.E.	Wald Chi-square	*P* value	Odds Ratio	Lower 95%	Upper CI
Ratio D/TRM	0.243	0.103	5.534	0.019	1.275	1.041	1.560
Days of menstruation	0.150	0.064	5.540	0.019	1.162	1.025	1.316
WBCs			6.133	0.047			
WBCs (1)	1.113	0.477	5.441	0.020	3.043	1.195	7.750
WBCs (2)	0.797	0.444	3.227	0.072	2.219	0.930	5.295
Fibrinogen	0.350	0.419	0.697	0.404	1.419	0.624	3.225

S.E.: Standard Error; 95% CI: 95% Confidence Interval

### Apparent performance and validation of the Nomogram

**Fig. 6:**
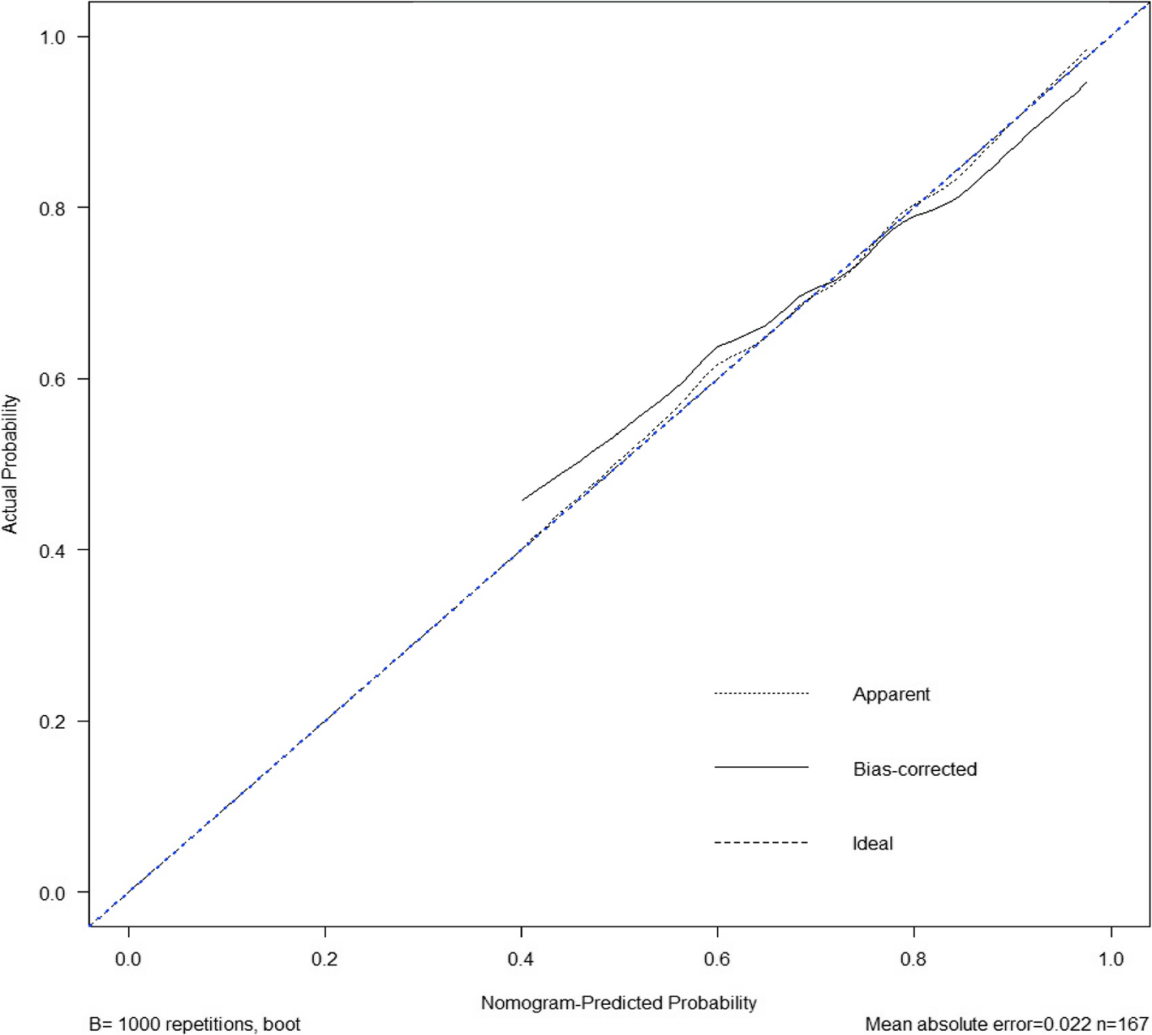
The nomogram-predicted probability. The nomogram yielded a C-index of 0.718 according to internal validation. The x-axis represents the predicted non-class-A healing probability. The y-axis represents the actual non-class-A healing rate. The diagonal dotted line represents an ideal model of prediction. The solid line represents a closer fit of the nomogram to the diagonal dotted line

The calibration curve of the nomogram for the probability of non-class-A healing in CSD patients showed adequate agreement between observation and prediction in the training cohort. The Hosmer-Lemeshow test presented a non-significant statistic (*P* = 0.976). This result represented no departure from a perfect fit. The C-index for the prediction model was 0.718 for the training cohort. The calibration curve depicted calibration of the model in terms of the agreement between the observed outcome of non-class-A healing and predicted risk of non-class-A healing. Furthermore, the prediction nomogram yielded a C-index of 0.718 according to internal validation of the nomogram (Fig. 6).

### Clinical use

**Fig. 7:**
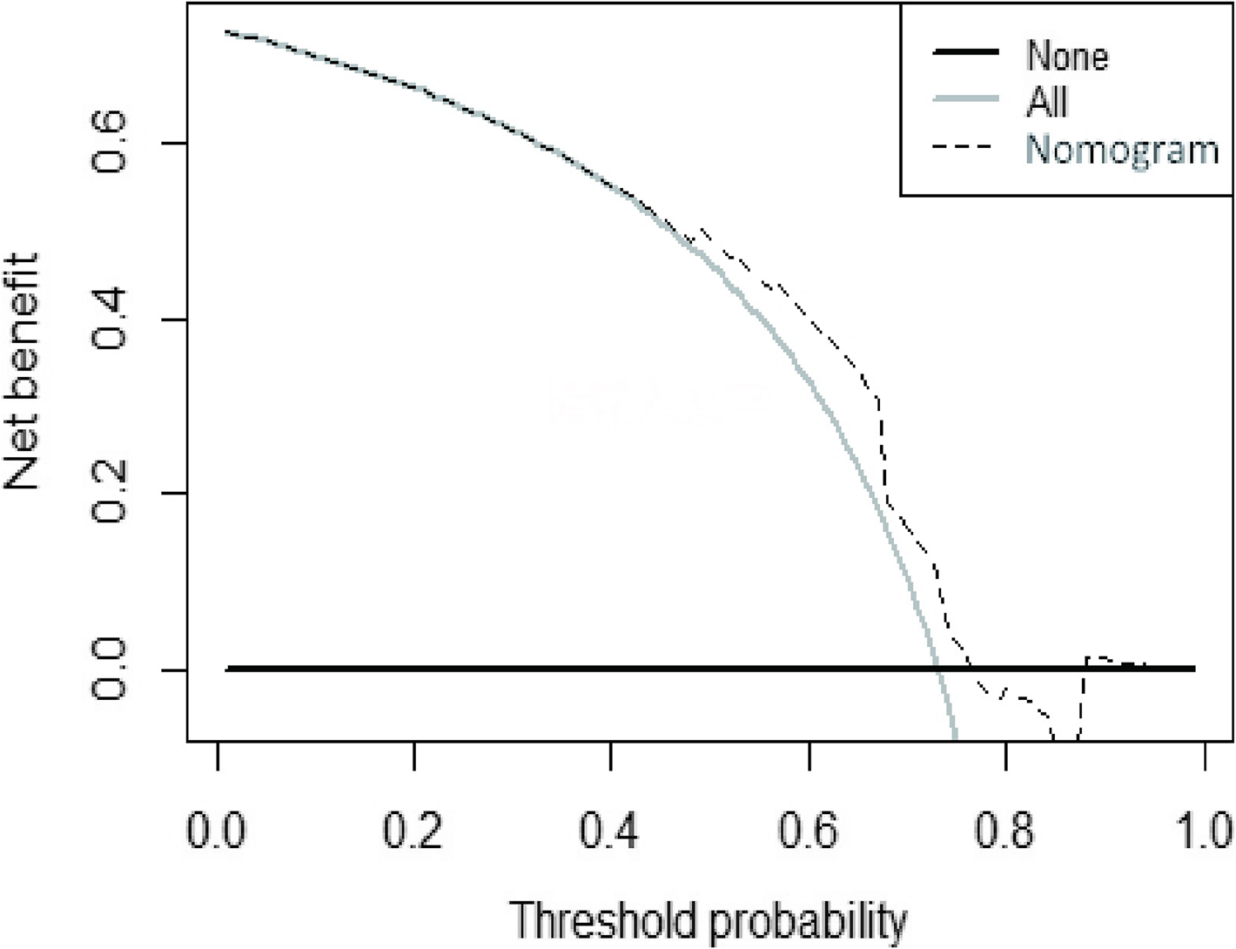
Decision curve for the nomogram. The y-axis measures the net benefit. The dotted line represents the nomogram. The gray line represents the assumption that all patients have cesarean section diverticulum. Thin black line represents the assumption that no patients have cesarean section diverticulum

The decision curve analysis for the nomogram is presented in Fig. 7. The decision curve demonstrated that using the nomogram to predict non-class-A healing added more benefit than either the treat-none scheme or treat-all-patients scheme. The y-axis represents the net benefit. The dotted line represents the nomogram, and the gray line represents the assumption that all CSD patients had non-class-A healing. The black line represents the assumption that no CSD patients had non-class-A healing. The net benefit was comparable within this range based on the nomogram. (Net benefit was defined as the proportion of true positives minus the proportion of false positives, weighted by the relative harm of false-positive and false-negative results [31, 32].) With the nomogram, we can provide individual treatment to the patients.

## Discussion

Cesarean section diverticulum (CSD) is a gynecological disease that leads to postmenstrual uterine bleeding, fluid collection in the wound pouch, chronic pelvic pain, dysmenorrhea and secondary infertility. It can also increase the risk of uterine rupture and cesarean scar pregnancy. However, until now, there have been no clinical guidelines for the preoperative prediction of patient outcomes for surgical treatment of cesarean section scar diverticulum. Vaginal repair of CSD is becoming a more common treatment compared to laparoscopic surgery and oral contraception. In the present study, we developed and validated a personalized nomogram for estimating preoperative prediction of non-class-A healing in patients with CSD. The nomogram incorporated four baseline items of the depth/TRM ratio, number of menstruation days before surgery, WBCs and fibrinogen. It is typically rare to use MRI for CSD diagnosis. Fiocchi reported that 3 T-magnetic resonance diffusion tensor imaging was better than transvaginal ultrasound for evaluating the thickness of the scar [16]. Thus, to detect the depth/TRM ratio for CSD, we used uterine contrast-enhanced MRI, which was more accurate than transvaginal ultrasound. With magnetic resonance imagings, we can measure the size of CSD multidirectionally and objectively, avoiding subjective bias which was a result of different level of sonographers and the quality of ultrasound machines. The duration of menstruation was related to a chronic inflammatory reaction, which was also associated with uterine wound healing. The longer chronic inflammation lasted, the harder the CSD was to treat. Thus, we used WBC as an inflammatory marker and fibrinogen as a coagulation function marker to develop a prediction model for evaluating surgical treatment outcomes for CSD patients.

The nomogram successfully stratified CSD patients according to their risk of non-class-A healing. To the best of our knowledge, this is the first study to give a quite practicable and convenient nomogram in the preoperative detection of non-Class-A healing with CSD patients. Thus, the easily available variables that we constructed could act as a convenient marker to predict non-class-A healing in Chinese patients with CSD. We hope to evaluate the effctiveness of surgery in our outpatientclinic with this nomogram. When we estimate a non-class-A healing of the patient, we tend to give conservative treatments such as oral contraceptive, gonadotropin-releasing hormone agonist (GnRHa). We aim to offer an adequate therapeutic method to those patients suffered with the most minimal hurt and the least cost.

However, this study had some limitations. First, in the training cohort, all patients were Chinese. Our analysis did not include people of any other race. Second, the research was conducted in a single hospital and was not a randomized controlled study that contained a large sample size. Furthermore, all patients did not use oral contraception after vaginal repair surgery, which could have suppressed luteinizing hormone (LH) and follicle-stimulating hormone (FSH), thereby causing temporary amenorrhea and low estrogen levels [17, 33]. Future work could include an evaluation of the nomogram in CSD patients treated with vaginal repair surgery and GnRHa. Thirdly, in our study, our patients from all over the china. There was no such a standardization for uterine suture in cesarean surgery. Moreover, only a few patients could provide us operation note. It was difficult for us to distinguish the different types of uterine suture. We will do more effort to find out if the art of the uterine suture influences niche’s severity. Finally, further comparison of other prediction models for CSD could be tested.

Despite the study limitations, this prognostic model was independently and internally validated with clinical datasets. We also applied a decision curve to evaluate whether the nomogram-assisted decision could improve CSD patient outcomes for clinical usefulness. According to our study, lower depth/TRM ratio, fewer number of menstruation days before surgery, lower level of WBC, higher level of fibrinogen indicate better progonisis. This novel method helped to predict clinical outcomes based on the threshold probability, and the net benefit could be derived. In the current study, the decision curve demonstrated that using the nomogram to predict non-class-A healing added more benefit than either the treat-none scheme or treat-all-patients scheme.

## Conclusions

This study demonstrated that an independently validated nomogram that combined both MRI scan results and clinical factors could be used to conveniently predict non-class-A healing in CSD patients.

## Data Availability

All patients signed written informed consent to participate in this study. The authors agreed to provide copies of the appropriate documentation if requested.

## Abbreviations

CSD: C-section scar diverticulum
C-section: Cesarean section
D/TRM ratio: Depth/ the thickness of the remaining muscular layer ratio
FSH: Follicle-stimulating hormone
GnRHa: Gonadotropin-releasing hormone agonist
Hb: Hemoglobin
LH: Luteinizing hormone
MRI: Magnetic resonance imaging
TRM: Thickness of the remaining muscular layer
